# Anti-Inflammatory Effects of Adenine Enhance Osteogenesis in the Osteoblast-Like MG-63 Cells

**DOI:** 10.3390/life10070116

**Published:** 2020-07-19

**Authors:** Yu-Pin Chen, Yo-Lun Chu, Yang-Hwei Tsuang, Yueh Wu, Cheng-Yi Kuo, Yi-Jie Kuo

**Affiliations:** 1 Department of Orthopedic Surgery, Wan Fang Hospital, Taipei Medical University, Taipei 11696, Taiwan; 99231@w.tmu.edu.tw (Y.-P.C.); adslme123@gmail.com (Y.-L.C.); birdywithlove@hotmail.com (Y.W.); 2 Department of Orthopedic Surgery, Shuang Ho Hospital, Taipei Medical University, New Taipei 23561, Taiwan; tsuangyh@gmail.com; 3 Department of Orthopedic Surgery, School of Medicine, College of Medicine, Taipei Medical University, Taipei 11031, Taiwan; 4 Department and Graduate Institute of Biology and Anatomy, National Defense Medical Center, Taipei 11490, Taiwan

**Keywords:** adenine, IL-6, osteogenesis, osteoblast, inflammatory bone disease

## Abstract

Background: Adenine is a purine with a role in cellular respiration and protein synthesis. It is considered for its pharmacological potential. We investigated whether anti-inflammatory effect of adenine benefits on the proliferation and maturation of osteoblastic cells. Methods: Human osteoblast-like cells (MG-63) were cultured with adenine under control conditions or pre-treated with 10ng/mL of tumor necrosis factor-α (TNF-α) followed by adenine treatment. Cell viability was examined using dimethylthiazol diphenyltetrazolium bromide (MTT) assay. Expression of cytokines and osteogenic markers were analyzed using quantitative PCR (qPCR) and ELISA. Enzyme activity of alkaline phosphatase (ALP) and collagen content were measured. Results: TNF-α exposure led to a decreased viability of osteoblastic cells. Treatment with adenine suppressed TNF-α-induced elevation in IL-6 expression and nitrite oxide production in MG-63 cells. Adenine induced the osteoblast differentiation with increased transcript levels of collage and increased ALP enzyme activity. Conclusions: Adenine exerts anti-inflammatory activity in an inflammatory cell model. Adenine benefits osteoblast differentiation in normal and inflammatory experimental settings. Adenine has a potential for the use to treat inflammatory bone condition such as osteoporosis.

## 1. Background

Osteoporosis is a common bone disease, which is characterized by poor bone strength and bone loss. It contributes to bone fragility and in turn fracture. Osteoporosis affects an estimated 75 million people in Europe, USA and Japan and causes more than approximately 9 million fractures worldwide every year [1,2]. It is prevalent in postmenopausal women, attributable to estrogen deficiency-related imbalanced bone resorption. Osteoporosis has a remarkable social and economic cost with a projected increase rate of 25% [3,4,5]. Clinically available treatments for osteoporosis include use of anti-resorption agents, such as bisphosphonates and hormone replacement therapy [6]. Despite the clinical benefits of the treatments, they still have a host of possible adverse effects [7,8]. Recently, the role of inflammation in bone remodeling has gained much attention. Pathogenesis of osteoporosis is multifactorial which involves inflammation [9]. Pro-inflammatory cytokines, such as primarily TNF-α and interleukin (IL)-6, are known to contribute to bone resorption and impaired bone remodeling, leading to deteriorated bone architecture [10,11,12,13]. In postmenopausal osteoporosis, deficient estrogen activates T-cells to produce an excessive amount of TNF-α and in turn inhibits osteoblast activity [14]. Previous studies have indicated an inhibitory effect of TNF-α on osteoblast differentiation [15,16]. TNF-α has inhibited levels of bone matrix components such as collagens and alkaline phosphatase [16,17]. A variety of agents with anti-inflammatory property have been demonstrated to have therapeutic benefits to osteoporosis [16,18,19,20,21]. In addition to synthetic agents, it is of interest to scientifically explore and examine food ingredients that facilitate bone homeostasis without adverse effects in a setting of osteoporosis.

Adenine is a purine base that is found in DNA and RNA. It is synthesized in the liver in human and found in a variety of foods, such as brewer’s yeast. Biological compounds containing adenine base include DNA in protein synthesis, nicotinamide adenine dinucleotide (NAD) and flavin adenine dinucleotide (FAD) in metabolism, adenosine triphosphate (ATP) in cellular respiration. In addition, recent studies have reported pharmacological potential of adenine. Adenine has been shown to attenuate allergic responses [22,23]. It has been reported to exert anti-inflammatory activity in different experimental settings [24,25]. Adenine is shown to protect cells from hostile conditions including rat Purkinje cells and erythrocyte in whole blood [26,27,28]. It is of interest to investigate the pharmacological effects of adenine on the proliferation and maturation of osteoblasts in osteoporosis in aspect of inflammation that contributes primarily to the bone disorder. 

In this study, we examined the pharmacological activity of adenine in bone metabolism with emphasis on cytokine-induced inflammation. Modulation of pro-inflammation in TNF-α treated osteoblast-like cells was determined. Osteoblasts are known to synthesize and secrete several components of bone matrix, mainly alkaline phosphatase (ALP), osteocalcin and collagens, while osteoclasts are involved in bone resorption. Regulation of bone metabolism-related gene expression mediated by adenine in TNF-α treated osteoblast was evaluated, including ALP and collagen type I, whichare two bone matrix components synthesized and secreted by osteoblasts.

## 2. Results

### 2.1. Effects of Adenine on Viability of Osteoblast-Like MG-63 Cells

**Figure 1:**
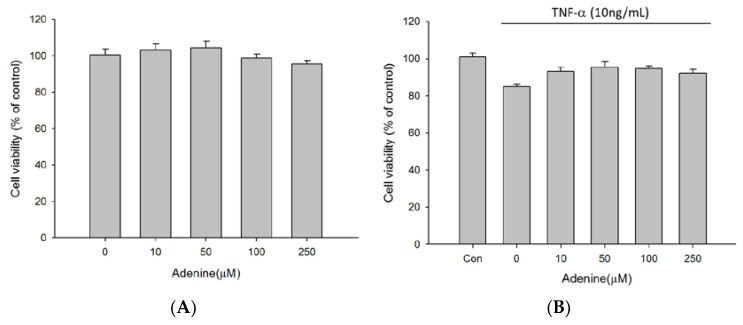
Effects of adenine on cell proliferation of MG-63 cells. (**A**) MG-63 cells were exposed to serial concentrations of adenine (0–250 µM/mL) for 24 h. (**B**) MG-63 cells were pre-cultured with TNF-α (10 ng/mL) and treated with serial concentrations of adenine (0–250 µM) for 24 h. Cell viability was determined using MTT assay. Data were expressed as mean ± SD.

To examine the influence of adenine on growth of osteoblasts, MG-63 cells were treated with serial concentrations of adenine (0–250 µM) in presence or absence of TNF-α for 24 h and the cell viability was evaluated using MTT assay. The results showed that adenine had no cytotoxicity on the growth of MG-63 cells (Figure 1A). Adenine had no significant influence on the slightly decreased viability of MG-63 cells exposed to TNF-α (10 ng/mL) (Figure 1B). 

### 2.2. Adenine Ameliorated TNF-α-Induced IL-6 Production in Osteoblast-Like MG-63 Cells

**Figure 2:**
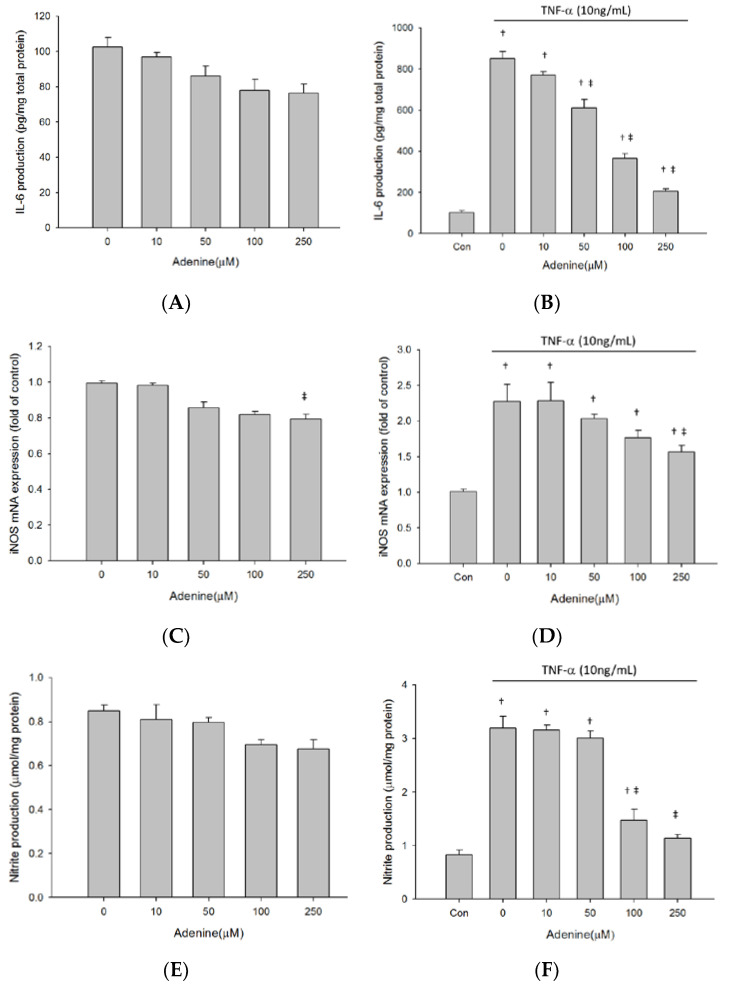
Effect of adenine on pro-inflammatory response in MG-63 cells. Cells were treated with or without 10ng/mL of TNF-α and cultured with serial concentrations of adenine (0–250 µM) for 24 h. (**A**,**B**) IL-6 production were determined by ELISA. (**C**,**D**) mRNA expression of iNOS upon TNF-α stimulation was examined using qPCR. (**E**,**F**) Nitrite production in culture medium of MG-63 stimulated with or without TNF-α was evaluated. Data were expressed as mean ± SD. ‡, *p* < 0.05 as comparing to 0 µg/mL; †, *p* < 0.05 as comparing to control (Con).

We next evaluated immunomodulatory effects of adenine on production of IL-6 in osteoblast-like cells upon stimulation of TNF-α. A low baseline expression of IL-6 was detected in the supernatant of untreated MG-63 cell culture. Adenine treatment dose-dependently decreased baseline IL-6 level in MG-63 cells (Figure 2A). Exposure to TNF-α led to a 10-fold increase in IL-6 production in MG-63 cells (Figure 2B). The TNF-α- induced elevation of IL-6 production was reduced in response to adenine treatment in MG-63 cells. We also investigated the influence of adenine on expression of inducible nitric oxide synthase (iNOS) that is upregulated by TNF-α in osteoblasts. The results showed that expression of iNOS was stimulated 2-fold by TNF-α, whereas adenine had no influence on naïve MG-63 cells (Figure 2C). Increases of iNOS expression were inhibited by adenine in dose-dependent manner (Figure 2D). The data of nitrite production in MG-63 cells supported the finding of reduction in iNOS expression elevated by TNF-α (Figure 2E,F).

### 2.3. Effects of Adenine on MG-63 Differentiation

**Figure 3:**
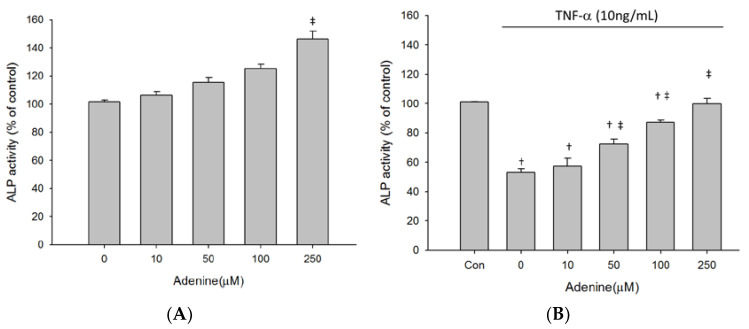
Adenine induced alkaline phosphatase (ALP) activity in MG-63 cells treated without (**A**) or with (**B**) stimulation of TNF-α. Cells were treated with serial concentrations of adenine (0–250 µM) for 24 h. Data were expressed as mean ± SD. ‡, *p* < 0.05 as comparing to 0 µg/mL; †, *p* < 0.05 as comparing to control (Con).

**Figure 4:**
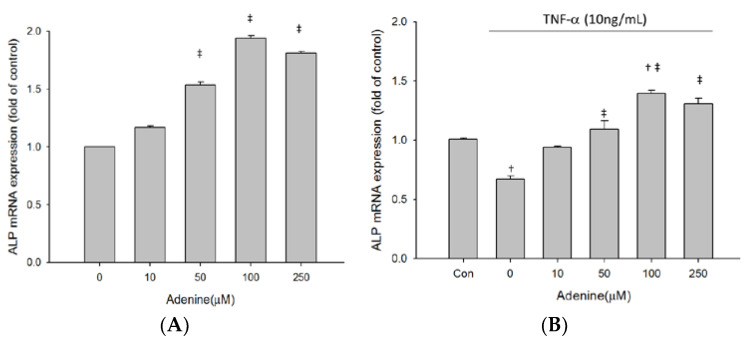
Adenine increased ALP mRNA expression in MG-63 cells treated without (**A**) or with (**B**) TNF-α. Cells were treated with serial concentrations of adenine (0–250 µM) for 24 h and mRNA expressions were determined by qPCR. Data were expressed as mean ± SD. ‡, *p* < 0.05 as comparing to 0 µg/mL; †, *p* < 0.05 as comparing to control (Con).

**Figure 5:**
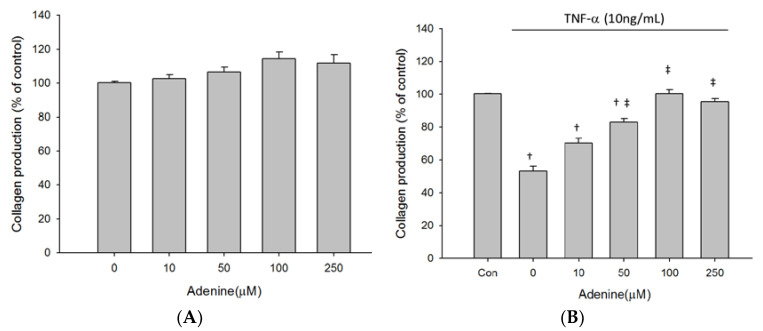
Effect of adenine on collagen synthesis in MG-63 cells treated without (**A**) or with (**B**) stimulation of TNF-α. Cells were treated with serial concentrations of adenine (0–250 µM) for 24 h and collagen synthesis in MG-63 cells was determined by ELISA. Data were expressed as mean ± SD. ‡, *p* < 0.05 as comparing to 0 µg/mL; †, *p* < 0.05 as comparing to control (Con).

**Figure 6:**
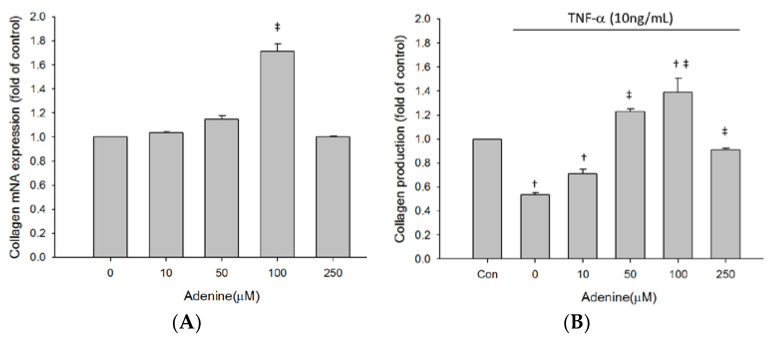
Adenine induced collagen type I mRNA expression in MG-63 cells treated without (**A**) or with (**B**) stimulation of TNF-α. Cells were treated with serial concentrations of adenine (0–250 µM) for 24 h and mRNA expressions were determined by qPCR. Data were expressed as mean ± SD. ‡, *p* < 0.05 as comparing to 0 µg/mL; †, *p* < 0.05 as comparing to control (Con).

As adenine treatment slightly restored inhibited proliferation of TNF-α-treated MG-63 cells, we next investigated whether adenine had effects on osteoblast differentiation by measuring expression and enzyme activity of ALP that is involved in osteoblast maturation. The results revealed that enzyme activity of ALP in MG-63 cells was increased by adenine treatment in a dose-dependent manner (Figure 3A). Exposure of MG-63 cells to TNF-α resulted in a significantly reduced ALP activity and the decrease of ALP activity was restored by adenine (Figure 3B). Adenine treatment led to increased mRNA expression of ALP and restored the reduced ALP expression in TNF-α-treated MG-63 cells (Figure 4A,B). In addition to ALP activity, we next evaluated the effects of adenine on collagenogenesis, which is an essential event in the differentiation of osteoblast. The results showed that adenine treatment increased the production of collagen type I in MG-63 cells at a concentration of 10 μg/mL (Figure 5A). TNF-α treatment (10 ng/mL) inhibited the production of collagen type I in MG-63 cells (p < 0.05) and the reduction was restored in response to adenine treatment in a dose-dependent manner (Figure 5B). Moreover, collagen level was upregulated in adenine-treated MG-63 cells at a concentration of 10 μg/mL (Figure 6A). Declined mRNA expression of collagen in TNF-α-treated MG-63 cells was restored sharing a similar pattern with that of protein synthesis (Figure 6B).

## 3. Discussion

In this study, we studied the anti-inflammatory activity of adenine with emphasis on the proliferation and differentiation of osteoblast. We found that adenine exhibited favorable anti-inflammatory activity on TNF-α-induced productions of IL-6 and NO in MG-63 cells. Our data revealed that adenine benefited differentiation of osteoblast, with evidence of an elevated ALP activity and collagen levels. The anti-inflammatory effect of adenine is suggested to reduce deterioration in osteogenesis.

Osteoporosis characterized by deterioration in bone structure is considered as low chronic inflammatory disease. Several pro-inflammatory cytokines have been reported to deteriorating bone architecture in turn, including TNF-α and IL-6 [11,29,30,31]. TNF-α has been shown to stimulate IL-6 secretion in osteoblastic cells, which acts in turn on osteoclasts and bone resorption [32,33]. Clinical findings are suggestive of the role of IL-6 in pathologic bone resorption [34,35]. Anti-TNF-α treatment has been demonstrated to improve inflammatory-induced bone loss through reducing IL-6 production [36,37,38]. In this study, we found that adenine exhibited anti-inflammatory activity, showing suppression of TNF-α-induced IL-6 production in osteoblastic cells. A decreasing trend of IL-6 expression was correlated with increases in cell viability of MG-63 cells. The finding is consistent with the results of previous studies reporting the effects of TNF-α on proliferation of osteoblastic cells [16,33]. In addition to IL-6, TNF-α induces expression of iNOS, leading to excessive NO production and in turn cytotoxicity in osteoblastic cells [39,40,41]. We reported that adenine repressed TNF-α-induced inflammatory phenomenon in osteoblast-like cells by inhibiting iNOS expression. Suppression of NO production by adenine treatment was associated with restoration of cell viability disturbed in presence of TNF-α. Given the findings, it is suggested that adenine contributes to proliferation of osteoblastic cell through anti-inflammatory actions in the current experimental setting.

Osteoblast differentiation is divided into three stages: cell proliferation, matrix maturation and mineralization [42]. ALP is expressed in osteoblast cells and considered as a marker for their early differentiation [43]. We showed that adenine treatment increased ALP expression and activity in MG-63 cells, suggesting that adenine benefits the differentiation of osteoblasts in the early stage. In TNF-α-stimulated MG-63 cells, treatment with adenine restored the reduced ALP expression and activity. The finding implies the beneficial potency of adenine for inflammatory bone disease. Collagen is mainly produced by osteoblast in bone maturation [44,45,46]. We showed that adenine increased collagen synthesis and collagen content in MG-63 cells. Reduction of collagen production by TNF-α was restored by adenine treatment, suggesting that adenine potentially benefits maturation of osteoblast cells. The findings reveal the adenine potentially stimulates the osteoblastic differentiation and the potency to enhance ALP activity and collagenogenesis.

We demonstrated that adenine exhibits anti-inflammatory activity and in turn contributes to the differentiation of osteoblast cells. It is suggested that adenine has beneficial effects on osteoporotic condition via partial restoration of bone matrix components. More studies are required to reveal the underlying mechanism by which adenine benefits the differentiation and maturation of osteoblasts.

## 4. Materials and Methods

### 4.1. Cell Culture

Human osteoblast-like MG-63 cells (ATCC^®^ CRL-1427™, Rockville, MD, USA) were cultured in Dulbecco’s modified Eagle’s medium (DMEM, Sigma, St. Louis, MO, USA) supplemented with 10% fetal bovine serum (FBS, Gibco, Carlsbad, CA, USA) and 100 μg/mL penicillin/streptomycin (all Sigma, St. Louis, MO, USA) at 37 °C. 6 × 10^5^ cells were seeded in a 6-well culture plate and grown until 80% confluence. For treatments with adenine (Sigma), TNF-α-treated MG-63 cell model was prepared as previously described [16]. In brief, cells were pretreated with TNF-α (10 ng/mL) in 1% FBS DMEM for 16 h followed by incubation with adenine with serial concentrations in serum-free DMEM for 24 h. After treatments, resulting cells were washed with phosphate-buffered saline (PBS; pH 7.2) and collected for following analyses. The control group was MG-63 cells without any treatment.

### 4.2. Cell Viability Assay

Cell viability was measured using MTT assay. Cells were treated with TNF-α and/or adenine, followed by an incubation with (3-(4,5-Dimethylthiazol-2-yl)-2,5-Diphenyltetrazolium Bromide) (0.5 mg/mL) at 37 °C for 4 h. The number of viable cells was directly proportional to the production of formazan determined by measuring the absorbance at 570 nm.

### 4.3. Alkaline Phosphatase Activity Assay

1 × 10^5^ of cells were seeded for each well of 24-well plates and incubated for 16–20 h, followed by exposure to adenine at various concentrations for 72 hr. After removing medium, remaining cells were lysed by freeze-thaw method and centrifuged at 15,000× g, 4 °C for 5 min. The resulting supernatant was subject to ALP activity assay. 20 μL of cell lysate was mixed with 100 μL Tris-glycine buffer (pH 10.3) and 100 μL of p-nitrophenyl phosphate (Sigma), followed by incubation at 37 °C for 30 min and adding 50 μL of 3 M NaOH. An absorbance reading of 405 nm was performed using microplate reader.

### 4.4. RNA Extraction, RT-PCR and Quantitative PCR (qPCR)

Total RNA of cell after treatments was extracted using Trizol (Sigma, Milpitas, CA, USA), according to the manufacturer’s protocol. mRNA levels were measured by qPCR amplification with designed primer pairs listed in Table 1 using the ABI PRISM 7700 sequence detection system (Applied Biosystems, Foster City, CA, USA). For mRNA quantitation, FastStart Universal SYBR Green Master (Roche Applied Science, Penzberg, Germany) was used. The threshold cycle numbers were calculated using the ΔΔCT relative value method and normalized to β-actin. Each qPCR reaction was repeated at least three times. 

### 4.5. ELISA

Collagen type I and IL-6, produced by osteoblast cells, were measured using ELISA. After treatments, culture media were collected and level of IL-6 was measured using Human IL-6 Quantikine ELISA Kit (R&D system, Minneapolis, MN, USA). Levels of collagen type I were assessed using Procollagen-C ELISA kit (Metra Biosystems, Santa Clara, CA, USA).

### 4.6. Nitric Oxide Assay

Griess reaction was carried out to measure nitric oxide concentration in cell culture medium. In brief, culture medium of cell treated in presence or absence of TNF-α, followed by incubation with adenine was collected and mixed with Griess reagent in a ratio of 1:1 and absorbance at 540 nm was measured. The concentration of nitrite was measured using sodium nitrite (NaNO_2_) as a standard.

### 4.7. Statistical Analysis

The values were shown as means ± SD. All results were analyzed by a one-way analysis of variance (ANOVA), incorporating with a Duncan multiple-comparison test. A *p* values < 0.05 indicated statistical significance.

## Figures and Tables

**Table 1:** Primers used in the PCR.

IL-6	5′-TAGCCCTGAGAAAGGAGACATG-3′	5′-AGGCAAGTCTCCTCATTGAATC-3′
iNOS	5′-GCGGAGCGATGGGAAGCATG-3′	5′-CCCGAGCTCCTGGAACCAC-3′
Alkaline phosphatase	5’-CATATCGTGTCCAAACTCAGT-3’	5’-ATAAACCCCCTGTGAAGTTGCA-3’
Collagen Type I	5’-AACAGGAAGGGCCACGACAA-3’	5’-GCGGCACAAGGGATTGAC-3’
β-actin	5’-TGACGTGGACATCCGCAAAG-3’	5’-CTGGAAGGTGGACAGCGAGG-3’

## Abbreviations

TNF-α	tumor necrosis factor-α
ALP	Alkaline phosphatase
ATP	adenosine triphosphate
DNA	deoxyribonucleic acid
NAD	nicotinamide adenine dinucleotide
FAD	flavin adenine dinucleotide
DMEM	Dulbecco’s modified Eagle’s medium
FBS	fetal bovine serum
PBS	phosphate-buffered saline
ANOVA	one-way analysis of variance
iNOS	inducible nitric oxide synthase
SLE	systemic lupus erythematosus
IBD	inflammatory bowel disease
MTT	3-(4,5-Dimethylthiazol-2-yl)-2,5-Diphenyltetrazolium Bromide
ELISA	enzyme-linked immunosorbent assay
